# Application value of isotropic acquisition with simultaneous multi-slice diffusion weighted imaging in subacute early small artery occlusive cerebral infarction

**DOI:** 10.3389/fneur.2026.1774766

**Published:** 2026-03-16

**Authors:** Chaoliang He, Jie Yang, Yan Li, Ling Tang, Hongfu Dan, Chao Yuan, Jianquan Zhong

**Affiliations:** Department of Radiology, Zigong First People's Hospital, Zigong, China

**Keywords:** diffusion weighted imaging, echo planar imaging, isotropy, simultaneous multi-slice, small artery occlusive cerebral infarction

## Abstract

**Background:**

To compare the application value of conventional single-shot echo planar imaging (SS-EPI), simultaneous multi-slice single-shot echo planar imaging (SMS + SS-EPI), and simultaneous multi-slice segmented readout isotropic acquisition in diffusion weighted imaging (SMS + RESOLVE-ISO DWI) for subacute early small artery occlusive cerebral infarction.

**Methods:**

A retrospective analysis was conducted on 60 patients with subacute early small artery occlusive cerebral infarction, who were treated with SS-EPI-ISO DWI, SMS + SS-EPI-ISO DWI, and SMS + RESOLVE-ISO DWI sequence was used for 1.5 mm isovoxel acquisition. Two radiologists independently assessed the image quality, combining multi-planar reconstruction (MPR), 3-Dimensional fluid attenuated inversion recovery (3D-FLAIR), and apparent diffusion coefficient (ADC) images to evaluate the number of infarct lesions. And subjective and objective evaluations of the image quality were conducted. Objective evaluations include contrast, signal-to-noise ratio (SNR), time SNR (tSNR), contrast-to-noise ratio (CNR) and ADC. Friedman’s test was used to analyze image indicators, Weighted Kappa test was employed to assess the consistency of subjective score, and intraclass correlation coefficient (ICC) was used to analyze the consistency of measurement results.

**Results:**

The scanning times for SS-EPI-ISO DWI, SMS + SS-EPI-ISO DWI, and SMS + RESOLVE-ISO DWI were 3:06, 1:38, and 4:51 min, respectively. The number of infarcted lesions showed that both SS-EPI-ISO DWI and SMS + SS-EPI-ISO DWI detected 129 lesions, while SMS + RESOLVE-ISO DWI detected 123 lesions. Subjective evaluation showed that SMS + RESOLVE-ISO DWI was superior to the other sequences in terms of overall image quality, artifacts, and geometric distortions (*p* < 0.001). Objective evaluation revealed significant differences in contrast, SNR, and tSNR among the three sequences (*p* < 0.001), but no significant differences in CNR and ADC values (*p* > 0.05).

**Conclusion:**

SMS + SS-EPI-ISO DWI achieves image quality comparable to that of SS-EPI-ISO DWI while shortening the scanning time. Although its suppression effect on artifacts and geometric distortion is not as good as that of the SMS + RESOLVE-ISO DWI sequence, the clarity of lesion display is similar to that of the SS-EPI-ISO DWI sequence. Therefore, it is suitable for the diagnosis of subacute early small artery occlusive cerebral infarction.

## 1 Introduction

Small artery occlusive cerebral infarction, defined as small subcortical ischemic lesions in the blood supply area of perforating branch arteries, is a clinically prevalent type of infarction, accounting for 20 to 30% of all ischemic strokes (1). In small artery occlusive cerebral infarction, the area of blood supply from perforating arteries is smaller, affecting less brain tissue, and posing less harm to patients compared to other types of cerebral infarction. In most cases, patients with small artery occlusive cerebral infarction do not exhibit clinical symptoms. However, small artery occlusive cerebral infarction can disrupt some neural conduction, leading to mild cognitive impairment (2). In severe cases, when the infarction involves important brain tissue, clinical symptoms become more pronounced, similar to acute cerebral infarction (AIS) (3).

Diffusion weighted imaging (DWI) is sensitive to restricted diffusion movement of water molecules in the body and is of important value in the early identification of cerebral infarction (4). It has been widely used in the diagnosis of cerebral infarction. Standard axial DWI sequences with thicker slice thickness and fewer slices are prone to false-negative results (5), often failing to detect smaller infarct lesions. Some studies have used voxel-based isotropic acquisition to accurately reveal stroke subtypes and distribution, but the acquisition time is longer (6). Therefore, in clinical practice, utilizing advanced imaging techniques to rapidly and accurately identify small artery occlusive cerebral infarction is beneficial for selecting treatment options and improving patient prognosis. To achieve high spatial resolution scanning while reducing scanning time, the simultaneous multi-slice (SMS) technique has been proposed. SMS utilizes multi-frequency pulses to simultaneously excite multiple slices, allowing for the acquisition of thinner and more extensive images within the same time frame. And it can shorten the repetition time. The obtained data is the entire K-space data, achieving the goal of shortening the acquisition time (7). This technique has been widely used in clinical practice (8, 9).

This study explores the clinical application value of isotropic (ISO) acquisition with conventional single-shot echo planar imaging (SS-EPI), simultaneous multi-slice single-shot echo planar imaging (SMS + SS-EPI), and simultaneous multi-slice segmented readout (SMS + RESOLVE) DWI in the subacute early small artery occlusive cerebral infarction.

## 2 Materials and methods

### 2.1 Participants

**Figure 1:**
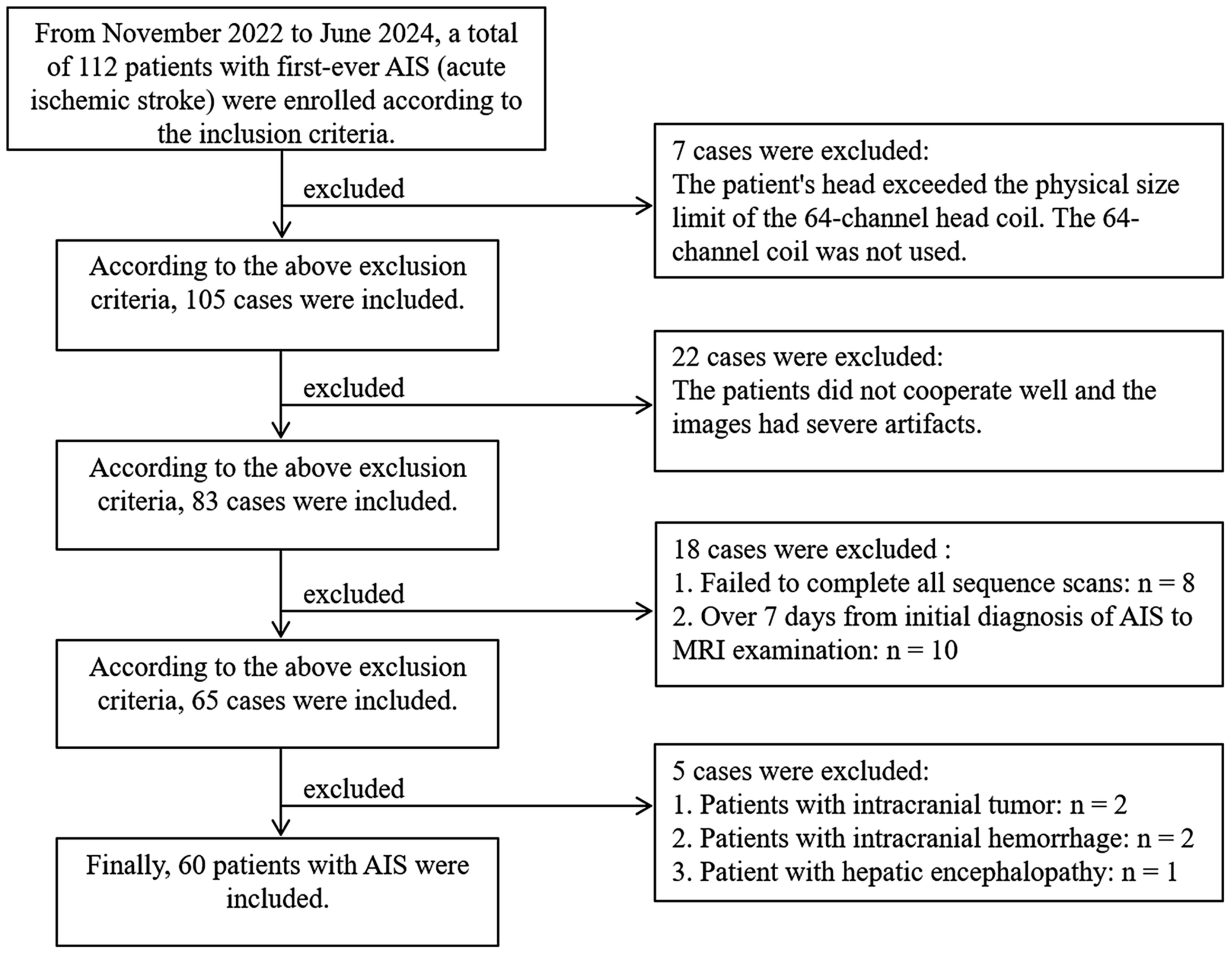
Flowchart of patient exclusion and inclusion process.

This study collected 112 patients with first-onset AIS diagnosed in the Department of Neurology of our hospital from November 2022 to June 2024. The diagnosis and onset time of the patient are determined by the neurologist based on the patient’s family statement, computed tomography angiography (CTA), computed tomography perfusion (CTP), and various laboratory tests. Inclusion criteria: (1) The condition meets the diagnostic criteria for ischemic stroke as outlined in the “Chinese guidelines for diagnosis and treatment of acute ischemic stroke 2018”; (2) After initial diagnosis, thrombolysis or interventional therapy was performed, followed by magnetic resonance imaging (MRI) examination. The time from initial diagnosis to MRI examination was less than 7 (3.129 ± 1.663) days; (3) The lesion exhibits high signal intensity on DWI (b = 1000s/mm^2^) images (with the maximum diameter of the infarct core <1.5 cm), and the apparent diffusion coefficient (ADC) of the corresponding region is low signal intensity; (4) All patients complete conventional head sequences, as well as SS-EPI-ISO DWI, SMS + SS-EPI-ISO DWI, and SMS + RESOLVE-ISO DWI sequence scan. Exclusion criteria: (1) Patients with MRI contraindications or whose heads exceed the physical size limit of the 64-channel head coil; (2) Patients with poor cooperation, resulting in severe image artifacts; or those who failed to complete the entire sequence scan; (3) Patients with comorbidities such as other mental disorders, cerebral hemorrhage, tumors, or other severe functional diseases; as shown in Figure 1.

According to the inclusion and exclusion criteria, 60 patients were finally included in this study (28 males, 32 females; age range: 28–93 years, mean ± standard deviation: 66.47 ± 11.61 years). The study was approved by the hospital Ethics Committee (Approval Number: Ethics (Research) No. 98, 2021). Written informed consent was obtained from all participants.

### 2.2 MRI acquisition

All participants underwent MRI on a Siemens 3.0 T MRI scanner (MAGNETOM VIDA, Siemens Healthcare, Erlangen, Germany) with a 64-channel head and neck combined coil. Imaging included conventional sequences (T2-weighted imaging, T1-weighted imaging, fluid attenuated inversion recovery (FLAIR), DWI) with 6 mm axial images and 3D-FLAIR sagittal images with 1 mm voxel size. Additionally, three 1.5 mm isotropic voxel DWI sequences were collected: SS-EPI DWI, SMS + SS-EPI DWI, and SMS + RESOLVE DWI. The scans of these three sequences were all performed in a random order. The scanning parameters are detailed in Table 1.

**Table 1:** MRI Sequences and Parameters.

Parameters/sequences	SS-EPI-ISO DWI	SMS + SS-EPI-ISO DWI	SMS + RESOLVE-ISO DWI
TR (ms)	9,100	4,400	3,060
TE (ms)	96	92	61/101
ES (ms)	0.96	0.96	0.36
Acquisition voxel (mm^3^)	1.5×1.5×1.5	1.5×1.5×1.5	1.5×1.5×1.5
Recon voxel (mm^3^)	0.75×0.75×1.5	0.75×0.75×1.5	0.75×0.75×1.5
FOV read (mm)	230	230	230
FOV phase (mm)	98.70%	100%	100%
Base resolution	154	154	154
Phase resolution	100%	100%	100%
b-value (s/mm^2^)	0,1,000	0,1,000	0,1,000
Readout segments	-	-	9
Averages	2, 5	2, 5	1, 3
Accel mode	GRAPPA	SMS	SMS
Acceleration factor PE	2	2	2
SMS factor	-	2	3
Slice	60	60	60
Thickness (mm)	1.5	1.5	1.5
Distance factor	30%	30%	30%
Diffusion mode	3-Scan Trace	3-Scan Trace	3-Scan Trace
TA (min)	3:06	1:38	4:51

### 2.3 Image analysis

#### 2.3.1 Subjective evaluation

Subjective evaluation was conducted by two experienced radiologists (a 7-year attending physician and a 15-year associate chief physician) on the SIEMENS (Syngo. Vida) workstation. They performed multi-planar reconstruction (MPR) (sagittal and coronal) reconstruction and evaluation of the images. Using a double-blind 5-point scoring system, they independently assessed the DWI sequence images with a b-value of 1000s/mm^2^ for artifacts, geometric distortions, lesion conspicuity, and overall image quality. The evaluation of each sequence was conducted at one-month intervals. Scoring criteria: Overall image quality, 1 point, poor: undiagnosable; 2 points, fair: generally meets diagnostic requirements; 3 points, slightly poor, meets diagnostic requirements, lesion contrast slightly poor, image resolution slightly poor, anatomical structure unclear; 4 points, good: meets diagnostic requirements, lesion contrast good, image resolution average, anatomical structure clear; 5 points, excellent: meets diagnostic requirements, lesion contrast very good, image resolution high, anatomical structure very clear. Artifacts and geometric distortions (sinuses, mastoid air cells, sella turcica region), 1 point, poor: severe artifacts and geometric distortions; 2 points, fair; significant artifacts and geometric distortions; 3 points, slightly poor: artifacts and geometric distortions present; 4 points, good: minor artifacts and geometric distortions; 5 points, excellent: no artifacts and geometric distortions. Lesion conspicuity, 1 point, poor: invisible; 2 points, fair: vaguely visible; 3 points, average, visible, slightly poor compared to surrounding tissue; 4 points, good: visible, clearly visible compared to surrounding tissue; 5 points, excellent: clearly visible.

#### 2.3.2 Objective evaluation

Two radiologists independently placed regions of interest (ROI) on images with b-values of 1,000 s/mm^2^ and ADC images from SS-EPI DWI, SMS + SS-EPI DWI, and SMS + RESOLVE DWI sequences, targeting the most prominent lesion layers and the corresponding normal tissues on the contralateral side. ROI were avoided in areas of tissue heterogeneity, and their sizes were adjusted according to the size of the lesion and the normal tissue on the contralateral side. The ROI were copied and pasted to ensure a one-to-one correspondence in size and position across the three sequences. Measurements were taken for the signal intensity (SI*_Lesion_*) of the lesion, signal intensity (SI*_normal_*) and standard deviation (SD) of the normal tissue on the contralateral side. Additionally, the background standard deviation (SD*_background_*) was measured at the same layer level (with a circular shape of 1.5 cm ± 0.5 cm) and averaged over two measurements. The content of this section has been revised as follows: Image contrast, signal-to-noise ratio (SNR), and contrast-to-noise ratio (CNR) were calculated, Equation 1–3. Furthermore, the time signal-to-noise ratio (tSNR) was determined for each sequence by dividing SNR by the acquisition time (10–12), (Equation (4).


$$\text{Contrast}=\frac{SI_{\text{Lesion}}}{SI_{\text{normal}}}$$ (1)

$$\mathrm{SNR}=\frac{SI_{\text{normal}}}{SD_{\text{backgound}}}$$ (2)

$$\mathrm{CNR}=\frac{SI_{\text{Lesion}}-SI_{\text{normal}}}{\sqrt{S{D^{2}}_{\text{Lesion}}+S{D^{2}}_{\text{normal}}}}$$ (3)

$$\;\text{tSNR}=\frac{\mathrm{SNR}}{\mathrm{Tim}e_{s}}$$ (4)

### 2.4 Statistical analysis

Statistical analysis was conducted using SPSS software (version 26.0) and MedCalc software (version 20.0). The normality of quantitative data was tested using the Shapiro–Wilk method. All quantitative parameters conforming to an approximate normal distribution were expressed as ±s, while those not conforming to a normal distribution were expressed as quartiles M (Q1, Q3). The subjective and objective evaluations of images were tested using the Friedman test. The consistency of subjective evaluations by two radiologists was assessed using the Kappa test, and the intra-class correlation coefficient (ICC) was used to compare the consistency of measurements (contrast, SNR, tSNR, CNR, ADC) between the two radiologists, represented by a 95% confidence interval; Kappa and ICC are between 0 and 1, and the closer they are to 1, the better the consistency between the two. The coefficient values are defined as follows (10, 11): <0.2 indicates poor consistency, 0.21 ~ 0.40 indicates moderate consistency, 0.41 ~ 0.60 indicates medium consistency, 0.61 ~ 0.80 indicates good consistency, and 0.81 ~ 1.00 indicates excellent consistency. *p* < 0.05 indicates statistical significance.

## 3 Results

### 3.1 Sequence scanning time

The scan times for SS-EPI-ISO DWI, SMS + S-EPI-ISO DWI, and SMS + RESOLVE-ISO DWI sequences are 3:06, 1:38, and 4:51 min, respectively.

### 3.2 DWI images reveal the number of infarct lesions

**Figure 2:**
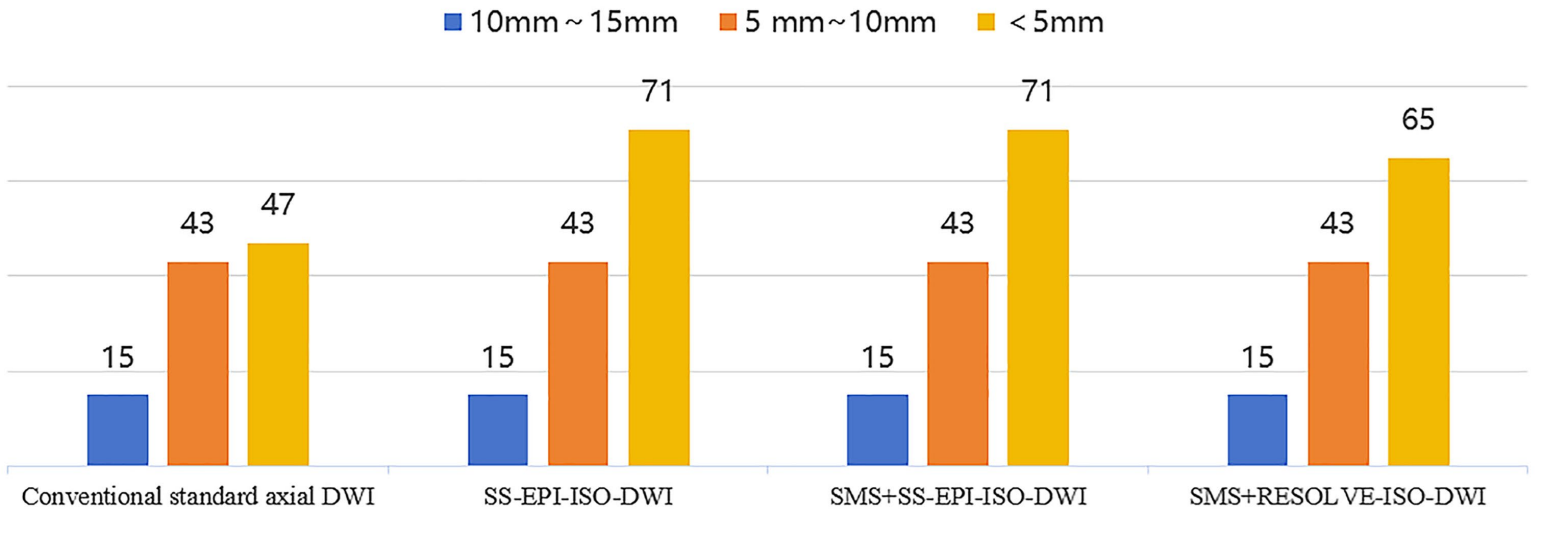
DWI image shows the number of infarcted lesions.

The conventional standard axial images displayed 105 lesions. The SS-EPI-ISO DWI images showed 129 lesions. The SMS + SS-EPI-ISO DWI images displayed 129 lesions. And the SMS + RESOLVE DWI images displayed 123 lesions, as shown in Figure 2.

### 3.3 Subjective evaluation

**Figure 3:**
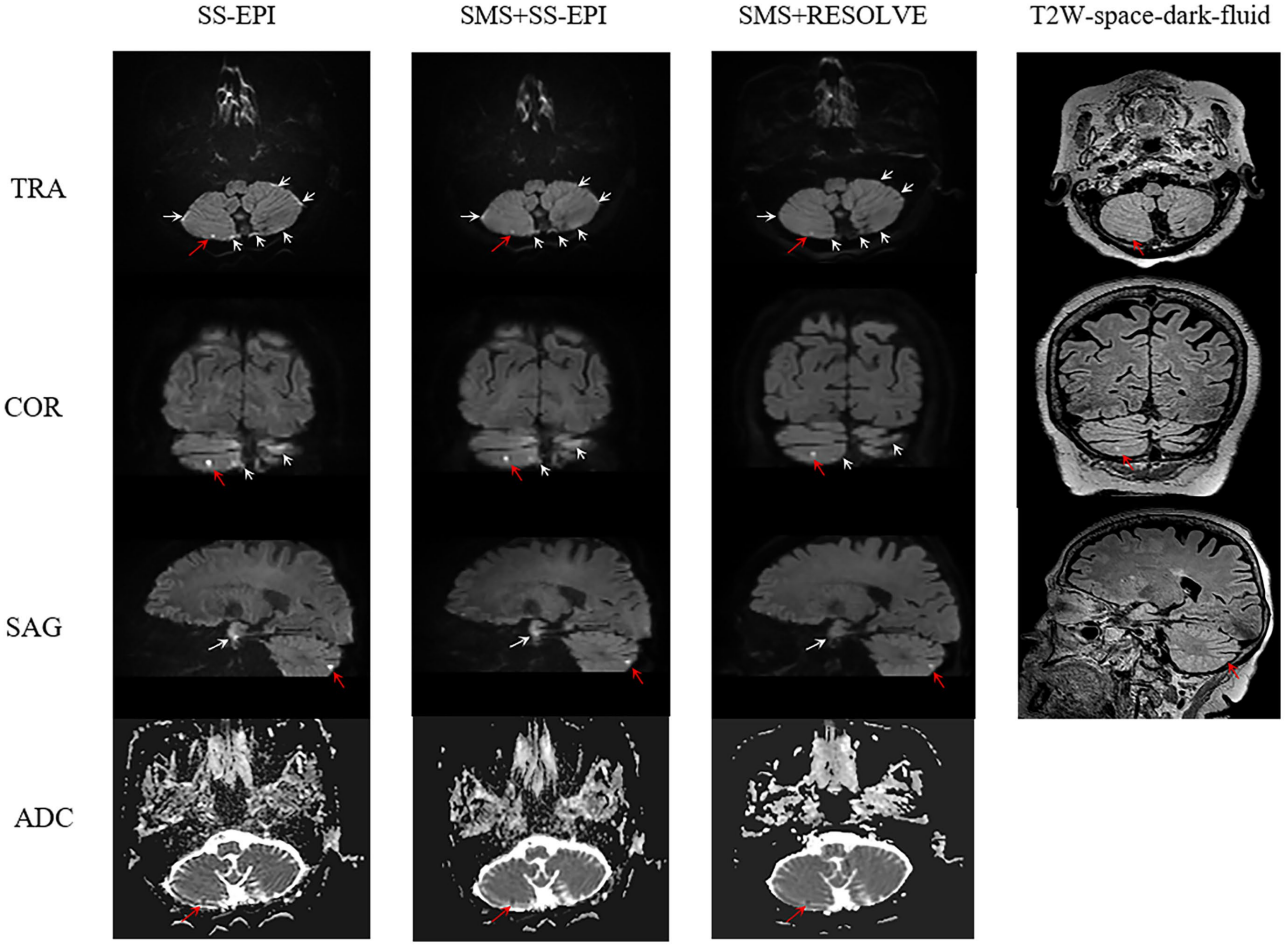
Female, 75 years old. On the SS-EPI-ISO DWI sequence (red arrow), punctate high signals in the right cerebellar hemisphere are indistinguishable from artifacts (white arrow). The apparent diffusion coefficient (ADC) shows low signals, with visibility score of 4 point, and artifacts and geometric distortions score of 2 point (white arrow), overall image quality score of 4 point. On the SMS + SS-EPI-ISO DWI sequence, faint punctate high signals are vaguely visible in the right cerebellar hemisphere, ADC shows low signals, with visibility score of 3 point, and artifacts and geometric distortions score of 3 point, Overall image quality score of 4 point. On the SMS + RESOLVE-ISO DWI sequence, a faint high signal is visible in the right cerebellar hemisphere. ADC shows low signal, with visibility score of 2 point, and artifacts and geometric distortions score of 2 point, overall image quality score of 5 point. The T2W-space-dark-fluid sequence reveals punctate high signals corresponding to the right cerebellar hemisphere.

**Figure 4:**
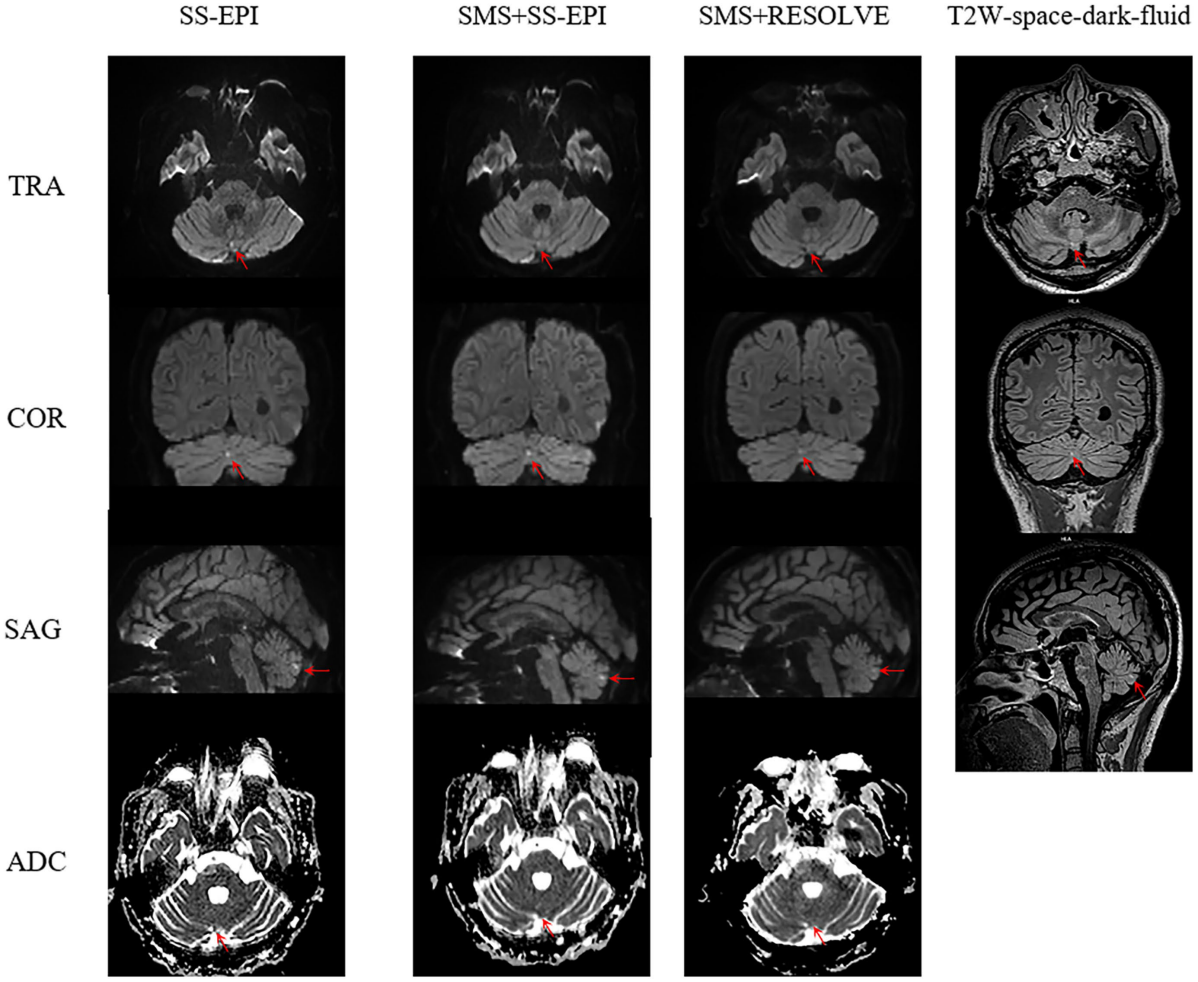
Male, 47 years old. SS-EPI-ISO DWI sequence (red arrow) reveals punctate high signal intensity in the vermis of the cerebellum, with low signal intensity on the apparent diffusion coefficient (ADC), scoring 4 in visibility. SMS + SS-EPI-ISO DWI sequence faintly shows blurry punctate high signal intensity, with low signal intensity on ADC, scoring 3 in visibility. The initial evaluation of the SMS + RESOLVE-ISO DWI sequence did not reveal any abnormal high signal, and retrospective comparison revealed a faint high signal in the vermis of the cerebellum with a visibility score of 1. T2W-space-dark-fluid sequence demonstrates corresponding punctate high signal intensity in the vermis of the cerebellum.

The three ISO sequence images meet the diagnostic requirements. The overall subjective scores (including overall image quality, artifacts and geometric distortions, and lesion saliency) given by the two radiologists exhibit strong consistency. The Kappa values are as follows: *κ* = 0.785 for SS-EPI-ISO DWI (95% CI: 0.638–0.931), κ = 0.718 for SMS + SS-EPI-ISO DWI (95% CI: 0.519–0.917), and κ = 0.766 for SMS + RESOLVE-ISO DWI (95% CI: 0.641–0.891), all with *p* < 0.001. There were statistically significant differences in the overall image quality scores, image artifacts and geometric distortions (in the sellar region, sinuses, and mastoid air cells) among the three ISO sequences (all *p* < 0.001). In pairwise comparisons, the overall image quality score of SMS + RESOLVE-ISO DWI was higher to both SS-EPI-ISO DWI and SMS + SS-EPI-ISO DWI sequences (both *p* < 0.001). There was no statistical significance between the overall image quality score of SS-EPI-ISO DWI and SMS + SS-EPI-ISO DWI (*p* = 1.000). There was a statistically significant difference in lesion visibility among the three ISO sequences (χ^2^ = 43.034, *p* < 0.001). Among them, the lesion visibility of SS-EPI-ISO DWI was superior to that of SMS + RESOLVE-ISO DWI (*p* < 0.001); the lesion visibility of SMS + SS-EPI-ISO DWI was superior to that of SMS + RESOLVE-ISO DWI (*p* = 0.001); there was no statistically significant difference in lesion visibility between SS-EPI-ISO DWI and SMS + SS-EPI-ISO DWI (*p* = 0.493), as shown in Table 2 and Figures 3,4.

**Table 2:** Comparison of subjective image scores for three sequences [point, M (Q1, Q3)].

Sequences/Subjective evaluation	Overall image quality (*n* = 60)	Artifacts (*n* = 60)	Geometric distortions (*n* = 60)	DWI lesion conspicuity (*n* = 129)
SS-EPI-ISO DWI	4 (4, 5)	3 (3, 3)	3(3, 3)	5 (4, 5)
SMS + SS-EPI DWI	4 (4, 5)	3 (3, 3)	3(3, 3)	5 (4, 5)
SMS + RESOLVE-ISO DWI	5 (5, 5)	4 (3, 4)	4(3, 4)	4 (3, 5)
*χ* ^2^	50.813	94.964	86.621	43.034
*p*	<0.001	<0.001	<0.001	<0.001

### 3.4 Objective evaluation

#### 3.4.1 Consistency of results

The ICC of overall lesion contrast, SNR, tSNR, CNR, and ADC values of normal tissue and lesions across the three sequences were as follows: the ICC of contrast is 0.804 (95% CI: 0.686–0.874); the ICC of SNR is 0.603 (95% CI: 0.375–0.752); the ICC of tSNR is 0.679 (95% CI: 0.481–0.791), the ICC of CNR is 0.895 (95% CI: 0.827–0.932); the ICC of normal tissue ADC values is0.862 (95% CI: 0.781–0.801); and the ICC of lesion ADC values is 0.834 (95% CI: 0.707–0.890), all with *p* < 0.001.

#### 3.4.2 Results of quantitative measurement

The difference in contrast between the three ISO sequences was statistically significant (χ^2^ = 53.200, p < 0.001); specifically, the contrast of SS-EPI-ISO DWI and SMS + SS-EPI-ISO DWI was higher than SMS + RESOLVE-ISO DWI (*p* < 0.001). There was no statistically significant difference in contrast between SS-EPI-ISO DWI and SMS + SS-EPI-ISO DWI (*p* = 1.000). The difference in SNR among the three ISO sequences was statistically significant (χ^2^ = 35.23, *p* < 0.001); specifically, the SNR of SMS + RESOLVE-ISO DWI was higher than both SS-EPI-ISO DWI and SMS + SS-EPI-ISO DWI (*p* < 0.001). There is no statistical difference in SNR between SS-EPI-ISO DWI and SMS + S-EPI-ISO DWI (*p* = 0.946). There was a statistically significant difference in tSNR among the three ISO sequences (χ^2^ = 64.933, *p* < 0.001); pairwise comparisons were also statistically significant (*p* < 0.05). The tSNR of SMS + SS-EPI-ISO DWI is higher than of SS-EPI-ISO DWI (*p* = 0.002); the tSNR of SMS + SS-EPI-ISO DWI is higher than of SMS + RESOLVE-ISO DWI (*p* < 0.001); and the tSNR of SS-EPI-ISO DWI is higher than of SMS + RESOLVE-ISO DWI (*p* < 0.001). There were no statistically significant differences in CNR, ADC values of normal tissue and lesion among the three ISO sequence images (*p* > 0.05), as shown in Table 3.

**Table 3:** Comparison of objective image scores for three sequences (*n* = 60).

Sequences/objective evaluation	Contrast ( $\bar{x}$ ±s)	SNR ( $\bar{x}$ ±s)	tSNR ( $\bar{x}$ ±s)	CNR [*M* (Q_1_, Q_3_)]	normal ADC value (x10^−3^ mm^2^/s) ( $\bar{x}$ ±s)	lesions ADC value (x10^−3^ mm^2^/s) ( $\bar{x}$ ±s)
SS-EPI-ISO DWI	1.982 ± 0.571	193.996 ± 62.001	1.102 ± 0.352	1.874 (1.381, 2.657)	0.755 ± 0.102	0.503 ± 0.109
SMS + SS-EPI DWI	1.942 ± 0.581	196.175 ± 70.955	1.453 ± 0.526	1.737 (1.256, 2.556)	0.757 ± 0.100	0.531 ± 0.092
SMS + RESOLVE-ISO DWI	1.606 ± 0.407	257.248 ± 72.299	0.884 ± 0.248	1.837 (1.367, 2.978)	0.764 ± 0.110	0.518 ± 0.109
*χ* ^2^	53.200	35.230	64.933	1.033	4.989	1.936
*p*	<0.001	<0.001	<0.001	0.597	0.590	0.083

## 4 Discussion

In this study, we employed ISO acquisition and compared the manifestations of three sequences, namely SS-EPI-ISO DWI, SMS + SS-EPI-ISO DWI, and SMS + RESOLVE-ISO DWI, in subacute early small artery occlusive cerebral infarction. The results indicated that compared to conventional standard DWI, ISO acquisition can enhance the detection of subacute early small artery occlusive cerebral infarction through MPR assistance. The SMS + SS-EPI-ISO significantly reduces acquisition time and yields DWI (b = 1000s/mm^2^) images that show no significant difference from conventional SS-EPI-ISO sequence. Although the suppression of artifacts and geometric distortions is inferior to that of SMS + RESOLVE-ISO DWI sequence, the lesion visibility is similar to that of SS-EPI-ISO DWI sequence.

DWI is the gold standard for detecting AIS. Clinically, conventional standard DWI of the head typically adopts a slice thickness of 5-6 mm due to scanning time considerations. Making it difficult to detect small cerebral infarction lesions (< 5 mm). Reports ^[5]^indicate that false-negative results are prone to occur in cases of ultra-acute stroke or small lesions. Therefore, more reliable methods are needed to improve accuracy and early identification of these lesions, which is beneficial for treatment plan selection and improving patient prognosis. Some studies have proposed using sagittal and coronal views to enhance the detection of brainstem infarctions (13–15). Our study employs high-resolution isotropic volume acquisition combined with MPR to detect the number of infarction lesions (<5 mm), which outperforms conventional standard 6 mm acquisition modes. Head 3D DWI has been proven feasible (16). There are also reports using 2.2 mm isotropic voxel acquisition for ischemic stroke subtype classification, but the acquisition time is longer (6). SMS acceleration mode employs multi-frequency excitation pulses to simultaneously excite multiple slices for acquisition. By separating the aliased images acquired from multiple slices, image quality is ultimately ensured while reducing acquisition time (7). In our study, using a 64-channel head and neck combined coil with SMS + SS-EPI-ISO DWI acquisition time of 1:38 min. It was possible to complete an examination of 60 slices with a thickness of 1.5 mm, and the image quality met diagnostic standards. Furthermore, the tSNR was superior to that of the other two sequences, thus improving time efficiency. Compared to conventional isotropic volume acquisition with the SS-EPI-ISO DWI sequence, the acquisition time was reduced by approximately 47%, and compared to the SMS + RESOLVE-ISO DWI sequence, the acquisition time was reduced by approximately 75%. There were no statistical differences in the CNR and ADC values of the images, which is consistent with previous research reports (17–19). Additionally, the consistency of measurement data between two radiologists was evaluated using ICC, confirming the reliability of the results.

The data in this group demonstrates that the SMS + RESOLVE-ISO DWI sequence excels in overall image quality, SNR, and the suppression of artifacts and geometric distortions compared to the SS-EPI-ISO DWI and SMS + SS-EPI DWI sequences. This reason is primarily attributed to the susceptibility distortion caused by B_0_ field inhomogeneity in the EPI sequence, which disrupts the diffusion image, manifesting mainly as geometric deformation and intensity variations. Intensity variations result from the accumulation or dispersion of pixel intensity due to pixel displacement, leading to bright or dark areas on the image. As the echo spacing (ES) increases, image deformation intensifies, and intensity variations become more pronounced (20). In our study, there was statistical significance in SNR among the three sequences, which is inconsistent with the findings of Xu (17) and Byeon et al. (19). There are multiple factors affecting SNR. We speculate that the inconsistency may be due to variations in the field of view (FOV) and matrix size in their studies. In our study, the FOV and matrix size were consistent across the three sequences. The RESOLVE sequence, by partitioning k-space into separate segments along the readout direction, reduces the ES from 0.96 ms to 0.36 ms. This shortens the effective echo time, improves the SNR, and reduces susceptibility artifacts and geometric distortion in the images (10). Multiple studies have confirmed that (17, 19, 21) RESOLVE can reduce geometric distortions and magnetic susceptibility artifacts in head applications. The SS-EPI DWI sequence has a longer repetition rime (TR), and the excessively long single sampling time leads to T2* effects, causing image blurring. However, the RESOLVE sequence has two TE values (imaging echo and naviator echo) corresponding to it. The naviator echo can correct large phase differences or nonlinear phases. And it can also utilize naviator echo information to read out some of the missing k-space data in Fourier space. Additionally, RESOLVE shortens the TR, reduces the single sampling time, and mitigates the image blurring effect caused by T2* effects, thereby enhancing overall image quality. Nevertheless, due to the segmented readout and subsequent integration to complete the entire k-space, the imaging time increases with the number of k-space segments (10, 12). Despite setting the readout segments to 9 and the SMS factor to 3 for the SMS + RESOLVE-ISO DWI sequence during scanning. It still takes about 5 min, which is why the tSNR of the SMS + RESOLVE-ISO DWI sequence is lower than that of the other two sequences.

The lesion conspicuity and contrast of the SMS + RESOLVE-ISO DWI sequence were inferior to those of the SS-EPI-ISO DWI and SMS + SS-EPI DWI sequences. This difference may be attributed to the higher temporal efficiency of EPI scanning, resulting in a higher tSNR for EPI. This is primarily due to EPI’s more efficient k-space coverage and the absence of additional navigators. The readout segmented RESOLVE sequence has a TE (imaging Echo) time of 61 milliseconds, which is shorter than the TE time of the EPI sequence (96 milliseconds, 92 milliseconds). A shorter TE leads to reduced T2 contrast on DWI images. In subacute infarct lesions, lesion visualization heavily depends on the “T2 shine-through” effect. SMS + RESOLVE-ISO DWI, with its shorter TE, significantly reduces the degree of T2 weighting, thereby diminishing the contrast between the lesion and background, resulting in lower contrast and conspicuity for the RESOLVE sequence (21). Although the subjective image quality score of the SMS + RESOLVE-ISO DWI sequence was superior to that of the SS-EPI-ISO DWI and SMS + SS-EPI DWI sequences, the protocol settings used in this study did not enhance the visual salience of infarct lesions. RESOLVE-ISO DWI Due to its relatively longer acquisition time, its widespread clinical application faces considerable obstacles. While there are few independent studies specifically comparing RESOLVE-DWI and conventional single-shot EPI (SS-EPI) in the hyperacute phase (<24 h), the RESOLVE sequence significantly reduces T2* blurring and geometric distortion through segmented readout, improving image quality, which is highly beneficial for stroke assessment requiring high resolution. In the hyperacute phase, the ADC value is a crucial indicator for diagnosing the ischemic lesions. Research has shown a high consistency in ADC measurements between RESOLVE and conventional SS-EPI sequences (22). For hyperacute stroke patients, who are often restless and require a very short scan time, SS-EPI remains the sequence of choice in most centers due to the slightly longer acquisition time of RESOLVE, especially when using high-resolution parameters. Limitations of the RESOLVE sequence have been reported for cholesteatomas (<3 mm) (23), potentially leading to false negatives or missed detection of these lesions. In our study, the SMS + RESOLVE-ISO DWI sequence missed the detection of six infarcts smaller than 5 mm compared to the long-TE EPI sequence. This indicates that the SMS + RESOLVE-ISO DWI protocol we used also has certain limitations in detecting small (<5 mm) infarcts (21, 23).

This study has some limitations. Firstly, the acceleration factors discussed in this article are relatively singular, without further analysis on whether other acceleration factors can further reduce scanning time under the condition of meeting diagnostic requirements. Additionally, only DWI images with b = 1000s/mm^2^ were analyzed, without comparative analysis of DWI images with b = 0 s/mm^2^.

## 5 Conclusion

Under the parameter settings of this study, SMS + SS-EPI-ISO can significantly shorten the acquisition time and can obtain DWI (b = 1000s/mm^2^) images that have no significant difference from the conventional SS-EPI-ISO sequence. Although the suppression of artifacts and geometric distortion is not as good as that of the SMS + RESOLVE-ISO sequence, the significance of the lesion is similar to that of the SS-EPI-ISO sequence. In this study, compared with the long-TE EPI, SMS + RESOLVE-ISO has a shorter TE and thus has insufficient T2-weighted weight. Therefore, it does not have an advantage in the display and detection of subacute cerebral infarction.

## Data Availability

The raw data supporting the conclusions of this article will be made available by the authors, without undue reservation.
